# Developing Inclusive Sports and Recreational Programs for Learners with Disabilities in Rural Limpopo: Barriers, Facilitators, and Impact on Well-Being

**DOI:** 10.3390/ijerph22121855

**Published:** 2025-12-12

**Authors:** Khodani Nemaranzhe, Phumudzo Khangwelo Mulibana, Khuliso Matshovhana, Anzani Mululuma

**Affiliations:** Department of Biokinetics, Recreation and Sport Science, Faculty of Health Science, University of Venda, Thohoyandou 0950, South Africa; phumudzo.mulibana@univen.ac.za (P.K.M.); khuliso.matshovhana@univen.ac.za (K.M.); anzani.mululuma@univen.ac.za (A.M.)

**Keywords:** inclusive sports, learners with disabilities, rural education, well-being, Limpopo, social inclusion, South Africa

## Abstract

Inclusive sports and recreational programs are essential for enhancing the physical, social, and psychological well-being of learners with disabilities. In South Africa (SA), and particularly in rural provinces such as Limpopo, the development of such programs remains limited due to infrastructural, socio-economic, and attitudinal barriers. This study explored the barriers and facilitators influencing inclusive sports and recreational opportunities, as well as their impact on the well-being of learners with disabilities in rural Limpopo. A qualitative study design was employed in selected rural special schools. Data were collected through semi-structured interviews with educators (*n* = 5) and focus group discussions with leaners with disabilities (*n* = 25) of ages ranging from 10 to 18 years using purposive sampling. Thematic analysis was guided by the Social Model of Disability, Self-Determination Theory, and Ecological Systems Theory. Findings revealed key barriers, including inaccessible infrastructure, a lack of adaptive equipment, and social exclusion. Educators further highlighted inadequate training, limited resources, and inconsistent policy implementation. Facilitators included teacher support, family involvement, and community initiatives. Participation in inclusive sports was associated with improved confidence, happiness, social skills, and belonging among learners. The study concludes that inclusive sports programs hold transformative potential in rural contexts. Addressing infrastructural gaps, teacher capacity community engagement policy implementation is critical for sustainable inclusion.

## 1. Introduction

Inclusive sports and recreational programs play critical roles in promoting the holistic development of learners with disabilities, fostering physical health, social integration, and psychological well-being [1]. In South Africa, and more specifically in rural areas such as Limpopo, the implementation of these programs remains limited, often due to socio-economic challenges and infrastructural deficits [2]. Despite national policies advocating for inclusive education and equal opportunities, there is a notable gap in how these initiatives translate into actionable and sustainable programs for learners with disabilities in rural settings [3].

Learners with disabilities often experience exclusion from mainstream sports activities due to physical, social, and attitudinal barriers [4]. In rural Limpopo, these challenges are compounded by insufficient resources, a lack of trained personnel, and limited awareness about disability-inclusive practices [5]. The lack of accessible infrastructure and adaptive equipment further restricts participation, reinforcing marginalization and reducing opportunities for positive peer interactions and skill development [6]. Investigating these obstacles is crucial to designing interventions that are contextually relevant and responsive to the needs of these learners.

Conversely, various facilitators can support the development of inclusive sports programs, such as community involvement, policy support, and collaboration between schools and local organizations [7]. Community attitudes towards disability have been gradually shifting towards greater acceptance, particularly when families and educators actively advocate for inclusivity [8]. Additionally, government and nongovernmental organizations have initiated programs aimed at capacity building and resource provision in rural districts, signaling important opportunities for strengthening inclusive practices [9]. Understanding these enablers can provide a foundation for scaling successful inclusive sports initiatives.

The impact of participation in inclusive sports and recreational activities extends far beyond physical health, influencing psychosocial well-being by enhancing self-esteem, social skills, and a sense of belonging [10]. For learners with disabilities, positive engagement in such programs has been linked to improved mental health outcomes, reduced social isolation, and increased academic motivation [11]. However, empirical data specific to rural Limpopo remains scarce, and localized evidence is essential to tailor programs that effectively address the unique socio-cultural and infrastructural realities learners face.

Therefore, this study aims to fill the existing research gap by identifying the barriers and facilitators involved in developing inclusive sports and recreational programs for learners with disabilities in rural Limpopo. Moreover, it seeks to evaluate the impact of these programs on the learners’ overall well-being. The findings will provide a framework for context-specific program development and policy implementation. Ensuring that inclusive sports become a practical and sustainable avenue for empowerment and integration of learners with disabilities in rural South African contexts.

### Theoretical Framework

The theoretical framework for this study is grounded in the Social Model of Disability.

This model shifts the focus from the individual’s impairment to the societal barriers that hinder participation and inclusion [12]. This model argues that disability is largely constructed by environmental, social, and attitudinal obstacles rather than by physical or mental impairments alone. Applying this perspective to sports and recreational programs emphasizes the importance of removing barriers whether physical, communicational, or cultural to enable equal access and participation for learners with disabilities [13]. In rural Limpopo, the Social Model offers a critical lens for understanding how contextual factors such as lack of infrastructure, social stigma, and policy gaps create disabling conditions that must be addressed to foster inclusive sports environments.

Complementing the Social Model, the theory of Self-Determination [14] provides insight into how participation in sports and recreational activities can enhance motivation, autonomy, and psychological well-being among learners with disabilities. This theory posits that individuals have basic psychological needs for autonomy, competence, and relatedness, and the fulfillment of these needs promotes intrinsic motivation and overall well-being. Inclusive sports programs that are designed to support these needs by offering adaptive activities, fostering supportive peer relationships, and encouraging skill mastery can thus positively impact the self-determination and empowerment of learners with disabilities [15]. This theoretical approach aligns with the study’s focus on well-being outcomes resulting from program participation.

Furthermore, Bronfenbrenner’s Ecological Systems Theory [16] provides a holistic framework for examining the multiple layers of influence affecting learners with disabilities in rural Limpopo.

This theory highlights the interactions between the individual and various environmental systems, including the microsystem (school, family), mesosystem (interactions between these settings), exosystem (community resources), and macrosystem (cultural and policy contexts). Recognizing that learners’ experiences of inclusion in sports are shaped by factors operating at these diverse levels is crucial for developing effective and sustainable programs. Addressing barriers and leveraging facilitators requires coordinated efforts across these interconnected systems, making Ecological Systems Theory an essential component of this study’s framework.

Together, the three models form a comprehensive framework: the Social Model identifies the external barriers to inclusion; Self-Determination Theory addresses individual psychological empowerment through participation; and Ecological Systems Theory situates these within broader environmental contexts that shape and mediate these processes. This linkage supports designing inclusive, empowering, and contextually responsive sports and recreation programs in rural Limpopo.

## 2. Materials and Methods

### 2.1. Approach and Design

This study adopts a qualitative research approach to gain in-depth understanding of the barriers, facilitators, and impact of inclusive sports and recreational programs for learners with disabilities. A case study design was employed to explore the experiences of learners and educators from rural Limpopo. This design allows for detailed, contextualized insights into the complexities of program development and its effects on well-being [17].

### 2.2. Setting

This study was conducted in selected rural special schools within Limpopo province, South Africa. These schools are characterized by limited resources, infrastructural challenges, and a diverse population of learners with various disabilities. The setting provides relevant context for examining inclusion practices in sports and recreation under rural conditions.

### 2.3. Population, Sample and Sampling Techniques

The population for this study includes learners with disabilities aged 10 to 18 years enrolled in rural Limpopo special schools, as well as educators involved in or knowledgeable about sports and recreational programs for learners with disabilities. Limpopo province has six (6) special schools for learners with disabilities. Only 42 learners with different disabilities participate in different sports and recreation activities in their schools.

A purposive sample of approximately 30 participants was selected to capture varied perspectives. This was made up of 25 learners with disabilities, 5 educators involved in or knowledgeable about sports and recreational programs for learners with disabilities. The sample size is deemed sufficient for thematic saturation in qualitative inquiry [18].

Purposive sampling was used to select participants who have direct experience or involvement with inclusive sports programs.

### 2.4. Inclusion and Exclusion Criteria

Inclusion criteria for learners included: aged 10–18, diagnosed with a physical, sensory, or cognitive disability, and active or previously involved in school or community sports/recreational activities for more than a year. Educators must have at least one year of experience involved in sports and recreational programs for learners with disabilities. Participants unable to communicate effectively in English or local languages were excluded to ensure meaningful data collection. From the total population of learners from different special schools, only 25 learners were eligible to form part of the study. In contrast, 17 learners were excluded from this study.

### 2.5. Data Collection Instruments and Data Collection Procedure

Data was collected through semi-structured interviews and focus group discussions, guided by instruments developed in line with the study’s theoretical framework. Both the interview guide and the focus group discussion guide were developed by the authors, guided by the theoretical framework and the literature. The interview guide had questions focusing on 1. challenges in program delivery, 2. support for the program and 3. perceptions of inclusion. While the focus group guide was composed of questions such as 1. barriers to participation, 2. facilitators for inclusion and 3. suggestions for improvement. data collection took place after the ethical clearance was obtained from Tshwane University of Technology ethics committee (protocol code FCRE 2021/02/009(SCI) (FCPS 02) Date of approval: 16 April 2021. Further approval was obtained from the Department of Basic Education, Vhembe District of Limpopo Province. The semi-structured interviews were conducted with Teachers to gain in-depth individual perspectives, while the focus group discussions were held with learners to capture shared experiences and collective viewpoints. These approaches were designed to explore participants’ experiences, perceived barriers and facilitators, and the impact of program participation on well-being.

### 2.6. Data Analysis

The thematic analysis followed [19] framework (familiarization, generating initial codes, searching for themes, reviewing themes, defining, and naming themes). Familiarization with the Data: Researchers immerse themselves in the raw data by listening to recordings, reading transcripts, and noting initial ideas and patterns relevant to inclusive sports programs, barriers, and facilitators for learners with disabilities in rural Limpopo.

Generating Initial Codes: Researchers coded the data using NVivo, isolating significant phrases, sentences, and paragraphs, and assigning codes to every transcript systematically, Meaningful segments of data were systematically coded. Codes represented features of the data that are of interest, such as identified barriers, facilitators, or well-being impacts experienced by learners. Searching for Themes: Codes were collated into themes that captured broader patterns or concepts within the data.

Reviewing Themes: Themes were refined by checking coherence within themes and distinctiveness between themes, ensuring they accurately reflect the dataset and research questions on inclusion and impact in rural contexts.

Defining and Naming Themes: Each theme was clearly defined and named to represent the essence of the data it captures, making them understandable and meaningful regarding inclusive sports development.

Producing the Report: The final step involved integrating themes into a coherent narrative that answers the research questions, providing evidence and interpretation on the barriers, facilitators, and well-being effects of inclusive programs for learners with disabilities.

## 3. Results

### 3.1. Summary of Themes

The learners’ focus group discussions revealed several key themes related to participation, inclusion, and well-being. These themes are summarized in Table 1, which outlines the main issues raised by the participants

### 3.2. Barriers to Participation

All learners *n* = 25 participated in the focus group discussion and the majority of Learners frequently identified physical obstacles as a primary barrier to participating in sports activities. Of all the participants (*n* = 25), a majority highlighted the lack of adapted equipment and sports facilities that do not accommodate their specific needs, such as wheelchair-accessible fields or sensory-friendly environments. Additionally, feelings of exclusion, either due to social stigma or inadequate support, were commonly expressed. One learner shared, “*The fields are not made for wheelchairs, so sometimes I just watch instead of playing.*” This shows how environmental and social barriers restrict active involvement.

### 3.3. Social Inclusion

The learners’ experiences with peer inclusion varied. Majority of learners reported positive relationships and invitation to join games, while, minority described exclusion and isolation in sports settings. Social attitudes played a critical role in their sense of belonging. For example, one participant said, “*Some friends include me, but others don’t want me to play with them.*” This highlights that social acceptance is uneven and impacts the learners’ motivation.

### 3.4. Facilitators for Inclusion

Support from teachers, family, and sometimes peers emerged as a key facilitator. When educators made efforts to adapt activities or personally encouraged participation, learners felt more included. Family encouragement also reinforced positive involvement outside school settings. A learner expressed, “*My teacher helps me join the games and makes sure I’m included.*” Such supportive actions help mitigate barriers.

### 3.5. Impact on Well-Being

Majority of participants reported multiple positive outcomes from participating in sports and recreation. These included increased self-confidence, happiness, improved social skills, and a greater sense of community. One learner stated, “*Playing makes me feel strong and happy, and I forget about my problems.*” This illustrates how sports can contribute to psychological and emotional well-being.

### 3.6. Suggestions for Improvement

Majority of Learners identified the need for more accessible facilities, specialized coaches trained in adaptive sports, and broader awareness campaigns to reduce stigma and promote inclusion. A common plea was: “*We need more equipment that fits our needs, like wheelchairs for sports and hearing aids for communication.*” Their suggestions reflect practical steps to enhance participation and enjoyment.

### 3.7. Results from Interviews with Teachers

Semi-structured interviews were conducted with five (5) teachers from selected special schools in Limpopo province who were purposively sampled based on their knowledge and involvement in sports and recreational activities for learners with disabilities. After the data was thematically analyzed, the following themes emerged:

Insights from teacher interviews further highlighted several structural and instructional challenges affecting inclusive sports and recreational programs. These findings are summarized in Table 2 below, which presents the key themes that emerged from the interviews.

### 3.8. Challenges in Program Delivery

Teachers pointed out major challenges such as insufficient resources, including a lack of specialized or adaptive sports equipment. Many also noted their own limited training in inclusive sport techniques, hindering their capacity to effectively deliver programs. One teacher remarked, “*We don’t have enough adaptive equipment here, so we often have to improvise or exclude some learners.*” Infrastructure constraints like inaccessible fields further complicated program execution.

### 3.9. Perceptions of Inclusion

While teachers generally acknowledged the importance of inclusive sports, their levels of understanding and commitment to it varied. Some expressed enthusiasm, but others were unsure about how to fully implement inclusive practices. For example, a teacher commented, “*Some teachers are not fully on board yet; they see it as extra work rather than necessary for all kids.*” This reflects gaps in attitudinal readiness.

### 3.10. Community and Policy Support

Interviewees noted that community involvement and government policies supporting inclusion exist but are inconsistently applied in practice. Support from local organizations varied, and policy directives often lacked the necessary funding or follow-up. A teacher said, “*Policy says inclusion is important, but we struggle with actual support and resources.*” This gap constrains program sustainability.

### 3.11. Positive Outcomes Observed

Teachers reported observing several benefits among participating learners, including enhanced social skills, better physical fitness, and increased motivation to engage in school life. One instructor shared, “*Sports help the kids open up and feel valued; you see them smile more and work better in class.*” These outcomes support the holistic value of inclusive programs.

### 3.12. Recommendations for Growth

Participants emphasized the urgent need for professional development workshops to build teacher capacity, increased funding for equipment, and partnerships with NGOs or community groups to broaden support. A teacher explained, “*We need workshops and more funding so we can properly train and get the right equipment for inclusive sports.*” These recommendations focus on addressing current resource and knowledge gaps.

## 4. Discussion

The study aimed at exploring the barriers and facilitators influencing inclusive sports and recreational opportunities, as well as their impact on the well-being of learners with disabilities in rural Limpopo.

The findings of this study align closely with the Social Model of Disability, which emphasizes that disability is largely a product of environmental and societal barriers rather than individual impairments [12]. Learners’ reports of physical obstacles, such as inaccessible fields and lack of adapted equipment, exemplify how these external barriers limit participation in sports and recreation. These barriers directly reinforce the disabling conditions highlighted by the Social Model, underscoring the need for systemic changes in infrastructure and resources to enhance inclusion. Similarly, the teachers’ accounts of inadequate resources and training deficiencies further confirm that environmental constraints continue to dominate the sports experiences of learners with disabilities in rural Limpopo.

The theme of social inclusion drawn from the data resonates with the Self-Determination Theory, which posits that individuals’ psychological needs for relatedness, competence, and autonomy are crucial for motivation and well-being [14]. Learners’ mixed experiences with peer acceptance and exclusion reflect challenges in fulfilling the basic need for relatedness, which can undermine intrinsic motivation to participate. However, support from teachers and family emerged as important facilitators, helping learners feel competent and connected within the sports setting. These supports demonstrate how nurturing environments, aligned with Self-Determination Theory, can enhance learners’ sense of empowerment and positive psychological outcomes, as evidenced by reported increases in self-confidence and happiness.

Bronfenbrenner’s Ecological Systems Theory [16] provides a valuable lens to understand the multilayered influences shaping the development and impact of inclusive sports programs. The study’s results illustrate how factors at multiple levels including microsystem supports like teachers’ encouragement and family involvement, ecosystem constraints such as limited community resources, and macrosystem elements like policy gaps interact to affect learners’ sports participation. Teachers’ recognition of policy commitments contrasted with practical limitations shows the disjuncture between ideal frameworks and lived realities. This confirms the ecological perspective that sustainable inclusion requires coordinated action across diverse systems to effectively address barriers and leverage facilitators.

Finally, the observed positive impacts of sports participation on learners’ well-being corroborate theoretical predictions and existing empirical evidence that inclusive sports foster psychosocial health and social integration [20]. Both learners and teachers described improvements in self-esteem, social skills, and motivation, highlighting sports as a critical avenue for promoting holistic development. However, these benefits appear contingent on addressing environmental and social barriers comprehensively. The findings indicate that addressing infrastructural gaps, building teacher capacity, enhancing community engagement, and improving policy implementation are critical to realizing the full potential of inclusive sports programs in rural Limpopo, aligning practice with the integrated theoretical frameworks guiding this study.

The study’s findings further demonstrated that inclusive sports and recreational programs serve as powerful tools for promoting disability rights, raising awareness, and fostering social change for people with disabilities. By creating environments that focus on abilities rather than limitations, these programs challenge stigma and discrimination, empowering learners to gain physical, social, and emotional benefits while developing essential life skills [21]. Education and training for teachers, coaches, and community workers based on international disability rights frameworks enable more effective inclusion, making sport a conduit for advocacy and empowerment [22]. Moreover, inclusive sports facilitate social integration and improve well-being, contributing to broader societal acceptance and equality for persons with disabilities [23].

Gender disparities are a critical area of concern, as girls and young women with disabilities face additional barriers shaped by cultural, social, and infrastructural factors, which limit their participation in inclusive sports programs. The study underscores the need for gender-sensitive policies and targeted efforts to create safe, accessible, and supportive sports environments that encourage active participation and leadership among female learners with disabilities [24]. Addressing both disability and gender inclusion simultaneously enhances empowerment and equity, advancing global disability rights agendas and fostering inclusive communities where every learner’s potential can be realized [25].

## 5. Limitations of the Study

The study was limited to a select group of rural schools located in Limpopo, which significantly reduces the generalizability of the findings to other regions within the country. Furthermore, the small, purposive sample utilized may not adequately represent the diverse experiences and needs of all learners with disabilities. The reliance on self-reported data could have introduced various forms of bias or inaccuracies, as individual perceptions may not fully capture the complexities of the educational environment.

Resource and accessibility challenges further constrained data collection efforts, restricting the researchers to easily reachable schools and potentially overlooking schools that serve different demographics or geographic areas. The limited opportunities for direct observation diminished the researchers’ capacity to understand real-time participation and engagement levels of learners, thus potentially skewing the interpretation of the data collected. Moreover, the exclusion of policymakers from the study curtailed insights into broader policy and systemic influences that are crucial for contextualizing the experiences of learners with disabilities in educational settings. Lastly, the cross-sectional design of the study captured experiences at a singular point in time, failing to account for potential changes and developments over time, which might affect the continuity and evolution of learners’ experiences in the educational system.

## 6. Practical Implications

The findings of this study have several important practical implications for enhancing inclusive sports and recreation among learners with disabilities in rural Limpopo:

Infrastructure Improvement: Schools and local authorities should prioritize the development of accessible sports facilities and provision of adapted equipment to remove physical barriers to participation.

Teacher Training and Capacity Building: Continuous professional development programs are needed to equip teachers with skills to deliver inclusive physical education and support learners with diverse abilities.

Policy Implementation: Education and sports policies promoting inclusion must be effectively translated into practice through monitoring, funding, and accountability mechanisms at school and district levels.

Community and Family Engagement: Strengthening collaboration between schools, families, and communities can foster supportive environments that encourage participation and acceptance of learners with disabilities.

Resource Allocation: Government departments and NGOs should allocate dedicated resources to inclusive sports initiatives, particularly in under-resourced rural areas.

Psychosocial Support: Programs should integrate emotional and social support components to build learners’ confidence, motivation, and sense of belonging in sports activities.

## 7. Conclusions

This study highlights the critical need for developing inclusive sports and recreational programs tailored to the unique context of learners with disabilities in rural Limpopo. The findings underscore that while learners experience significant barriers related to infrastructure, social acceptance, and resource limitations, supportive relationships and community involvement serve as important facilitators that enhance well-being and inclusion. Grounded in the Social Model of Disability, Self-Determination Theory, and Ecological Systems Theory, the study reveals that sustainable inclusion requires addressing systemic environmental barriers, fostering psychological empowerment, and coordinating efforts across multiple ecological levels. Ultimately, the successful implementation of inclusive sports programs holds transformative potential to improve physical, social, and emotional outcomes for learners with disabilities, provided that policy support, capacity building, and resource allocation are prioritized in rural settings.

## Figures and Tables

**Table 1:** Summary of Themes from Learners’ Focus Group Discussions (*n* = 25).

Theme	Description	Example Quotes
Barriers to Participation	Physical obstacles, lack of adapted equipment, and feelings of exclusion in sports activities	“*The fields are not made for wheelchairs*.”
Social Inclusion	Experiences of acceptance or exclusion among peers during sports and recreation	“*Some friends include me, but others don’t.*”
Facilitators for Inclusion	Support from teachers, family encouragement, and the availability of some adaptive activities	“*My teacher helps me join the games.*”
Impact on Well-being	Positive effects on self-confidence, happiness, and sense of belonging	“*Playing makes me feel strong and happy*.”
Suggestions for Improvement	Calls for more accessible facilities, specialized coaches, and awareness campaigns	“*We need more equipment that fits our needs.*”

**Table 2:** Themes of Teacher Interviews on Barriers, Facilitators, and Outcomes of Inclusive Sports and Recreational Programs in Rural Limpopo.

Theme	Description	Example Quotes
Challenges in Program Delivery	Limited resources, lack of training on inclusive sports, and infrastructural constraints	“*We don’t have enough adaptive equipment here*.”
Perceptions of Inclusion	Recognition of importance but varying levels of commitment or understanding of inclusivity	“*Some teachers are not fully on board yet*.”
Community and Policy Support	Importance of community involvement and government policies, but gaps in implementation	“*Policy says inclusion, but we struggle with support.*”
Positive Outcomes Observed	Noticeable improvements in learners’ social skills, fitness, and motivation	“*Sports help the kids open up and feel valued*.”
Recommendations for Growth	Need for training, funding, and partnerships to enhance sports and recreation programs	“*We need workshops and more funding for these programs*.”

## Data Availability

The data supporting the findings of this study, Developing Inclusive Sports and Recreational Programs for Learners with Disabilities in Rural Limpopo: Barriers, Facilitators, and Impact on Well-being, are available from the corresponding author upon reasonable request. Due to privacy and ethical considerations, which are essential to safeguarding the confidentiality of learners involved in this study. The data are not publicly accessible. All requests for access will be carefully reviewed to ensure compliance with ethical standards.

## Abbreviations

The following abbreviations are used in this manuscript:

FCRE	Faculty of Science Committee for Ethics
NGOs	Non Governmental Organizations
SA	South Africa
SCM	School Management Committees
